# Rod microglia: elongation, alignment, and coupling to form trains across the somatosensory cortex after experimental diffuse brain injury

**DOI:** 10.1186/1742-2094-9-247

**Published:** 2012-10-30

**Authors:** Jenna M Ziebell, Samuel E Taylor, Tuoxin Cao, Jordan L Harrison, Jonathan Lifshitz

**Affiliations:** 1 Department of Child Health, University of Arizona College of Medicine - Phoenix, Phoenix, AZ, USA; 2 Barrow Neurological Institute at Phoenix Children’s Hospital, Phoenix, AZ, USA; 3 Spinal Cord & Brain Injury Research Center, Department of Anatomy & Neurobiology, Department of Physical Medicine & Rehabilitation, University of Kentucky College of Medicine, Lexington, KY, USA; 4 Department of Biology and Biochemistry, University of Bath, Bath, England; 5 Neuroscience Program, Arizona State University, Tempe, AZ, USA; 6 Phoenix VA Healthcare System, Phoenix, AZ, USA

**Keywords:** Brain injury, Microglial rod cells, Rod microglia, Inflammation

## Abstract

**Background:**

Since their discovery, the morphology of microglia has been interpreted to mirror their function, with ramified microglia constantly surveying the micro-environment and rapidly activating when changes occur. In 1899, Franz Nissl discovered what we now recognize as a distinct microglial activation state, microglial rod cells (*Stäbchenzellen)*, which he observed adjacent to neurons. These rod-shaped microglia are typically found in human autopsy cases of paralysis of the insane, a disease of the pre-penicillin era, and best known today from HIV-1-infected brains. Microglial rod cells have been implicated in cortical ‘synaptic stripping’ but their exact role has remained unclear. This is due at least in part to a scarcity of experimental models. Now we have noted these rod microglia after experimental diffuse brain injury in brain regions that have an associated sensory sensitivity. Here, we describe the time course, location, and surrounding architecture associated with rod microglia following experimental diffuse traumatic brain injury (TBI).

**Methods:**

Rats were subjected to a moderate midline fluid percussion injury (mFPI), which resulted in transient suppression of their righting reflex (6 to 10 min). Multiple immunohistochemistry protocols targeting microglia with Iba1 and other known microglia markers were undertaken to identify the morphological activation of microglia. Additionally, labeling with Iba1 and cell markers for neurons and astrocytes identified the architecture that surrounds these rod cells.

**Results:**

We identified an abundance of Iba1-positive microglia with rod morphology in the primary sensory barrel fields (S1BF). Although present for at least 4 weeks post mFPI, they developed over the first week, peaking at 7 days post-injury. In the absence of contusion, Iba1-positive microglia appear to elongate with their processes extending from the apical and basal ends. These cells then abut one another and lay adjacent to cytoarchitecture of dendrites and axons, with no alignment with astrocytes and oligodendrocytes. Iba1-positive rod microglial cells differentially express other known markers for reactive microglia including OX-6 and CD68.

**Conclusion:**

Diffuse traumatic brain injury induces a distinct rod microglia morphology, unique phenotype, and novel association between cells; these observations entice further investigation for impact on neurological outcome.

## Background

Work published by renowned neuropathologist Franz Nissl described *Stäbchenzellen* (rod cells) in general paralysis of the insane [1]. These rod cells are now understood to represent a form of activated microglia, with a host of possible functional consequences [2]. By the early 20th century, their presence had been confirmed by others including Alzheimer, del Rio Hortega, Cerletti, and Ulrich, with detailed descriptions compiled by Achúcarro, who identified rod microglia in other diseases in both humans and animals (reviewed in [3]). These cells were shown to align with nerve cells and were depicted as strung-out, long, slim glial cells with rod-like nuclei placed perpendicular to the cortical surface [2].

Rod microglial cells have been principally portrayed in association with infections such as typhus, syphilis, and sleeping sickness, but the decline of these illnesses has all but ended further investigation. To date, the bulk of literature on rod microglia is limited to the morphology and possible association to neurons via historical monographs. More recent literature has identified these cells, albeit in isolation, in many neuropathological and experimental conditions including stroke, Alzheimer’s disease, and encephalitis [4-7]. Despite rod microglia being acknowledged, their role remains enigmatic.

After traumatic brain injury (TBI), microglial activation is consistently observed in patients [8-10], with many experimental models of focal and diffuse brain injury detailing altered microglia morphology [11-14]*.* Focal TBI typically results in the rapid accumulation of amoeboid microglia/macrophages within the cortical penumbra, with activated microglia surrounding the cortical contusion [13-15]. On the other hand, microglia activation has been noted after diffuse TBI in the corpus callosum, cortex, thalamus, and hippocampus with the number of CD68+ cells increasing from day 1 to day 28 post-injury [12,16]. Furthermore, it has been proposed that the preserved neuronal tissue after an insult like diffuse TBI is critical for the formation of rod microglial cells [4]; with extensive cellular damage or necrosis, only phagocytic microglia can be found.

The vast majority of microglial literature focuses on the ramified active or amoeboid morphologies, with no experimental investigation into the role of the rod morphology. Additionally, the rod microglial cells that have been described have been shown to be highly polarized, single cells. Here we describe these understudied cells in a new morphological phenomenon, where multiple cells are observed in a single trajectory, with their highly polarized processes seemingly connected; giving a coupled appearance. We have discovered these coupled rod microglial cells as a feature of midline fluid percussion injury (mFPI), an experimental model of diffuse traumatic brain injury. This communication focuses on the location of these cells as well as the architecture, which surrounds them. We found rod microglia to align perpendicular to the dural surface of the primary sensory cortex; most notable within the primary somatosensory barrel field (S1BF). As reported almost a century ago in other neurological conditions, the microglia align parallel and adjacent to the neural processes after mFPI.

## Materials and Methods

### Surgical preparation and diffuse brain injury

Adult male Sprague-Dawley rats (350 to 375 g) were subjected to midline fluid percussion injury (mFPI) consistent with methods described previously [17-20]. Briefly, rats were anesthetized with 5% isoflurane in 100% O_2_ prior to the surgery and maintained at 2% isoflurane via nose cone. Rats were placed in a stereotaxic frame and a midline scalp incision was made to expose the skull. A 4.8-mm circular craniotomy was performed (centered on the sagittal suture midway between bregma and lambda) without disrupting the underlying dura or superior sagittal sinus. An injury hub was fabricated from the female portion of a Luer-Loc needle hub, which was cut, beveled, and scored to fit within the craniotomy. A skull screw was secured in a 1-mm hand-drilled hole into the right frontal bone. The injury hub was affixed over the craniotomy using cyanoacrylate gel and methyl-methacrylate (Hygenic Corp., Akron, OH, USA) was applied around the injury hub and screw. The incision was sutured at the anterior and posterior edges and topical Lidocaine ointment was applied. Animals were returned to a warmed holding cage and monitored until ambulatory.

For injury induction, animals were re-anesthetized with 5% isoflurane 60 to 90 min after surgery. The dura was inspected through the injury-hub assembly, which was then filled with physiological saline and attached to the male end of the fluid percussion device (Custom Design and Fabrication, Virginia Commonwealth University, Richmond, VA, USA). As the rat’s reflexive responses returned, a moderate injury (1.9 to 2.0 atm) was administered by releasing the pendulum onto the fluid-filled cylinder. Animals were monitored for the presence of a forearm fencing response as well as the return of the righting reflex as indicators of injury severity [17]. Sham animals were connected to the FPI device, but the pendulum was not released. The injury-hub assembly was removed *en bloc*, integrity of the dura was observed, and bleeding was controlled prior to the incision being stapled. Moderate brain-injured animals had righting reflex recovery times of 6 to 10 min, and sham-injured animals recovered within 15 s. Surgical recovery was monitored postoperatively for 3 days, for which no overt differences (for example, weight, movement, grooming) were observed between animals. Animal experiments were conducted in accordance with NIH guidelines and approved by the University of Kentucky institutional animal care and use committee (IACUC). Measures were taken to minimize pain and discomfort.

### Immunohistochemistry for DAB stained sections

At designated time points (1, 2, 7, or 28 days; *n* = 3 per time point) post-injury or sham operation, rats were overdosed with sodium pentobarbital (200 mg/kg i.p.) and transcardially perfused with saline, followed by 4% paraformaldehyde in PBS. Following decapitation, the heads were stored in paraformaldehyde fixative solution containing 15% sucrose for 24 h, after which the brains were removed, placed in fresh fixative, and shipped for histological processing to Neuroscience Associates Inc. (Knoxville, TN, USA). Sixteen rat brains were embedded into a single gelatin block (Multiblock® Technology; Neuroscience Associates). Individual cryosections containing all the rat brains were mounted onto large glass slides (75 x 50 x 1 mm). These slides were then immunostained for ionized calcium binding adaptor molecule 1 (Iba1) to identify all microglia. The stained slides were photographed using an Olympus AX80 Automatic Research microscope with attached digital camera.

### Tissue preparation and fluorescent labeling

At 7 days post-injury (*n* = 6) or sham operation (*n* = 6), rats were given an overdose of sodium pentobarbital and transcardially perfused with 4% paraformaldehyde after a PBS flush. Brains were removed and cryoprotected in 30% sucrose. After freezing, brains were cryostat sectioned in the coronal plane at 20 μm and mounted onto gelatinized glass slides. For double-labeling with rabbit anti-Iba1 (1:2,000, WAKO, cat# 019919741) or mouse anti-Iba1/AIF (1:2,000, Millipore, cat# MABN92), six to twelve sections per animal were defrosted and washed in PBS containing 0.1% Tween-20 (PBST) thrice before being blocked in 4% v/v normal goat serum, 0.4% v/v TritonX-100 in PBS. Primary antibodies were added to 1% blocking solution (mouse anti-GFAP, 1:5,000, Millipore, cat# MAB360; mouse anti-neurofilament, 1:400, Sigma-Aldrich, cat# N0142; mouse anti-MAP2, 1:2,000, Sigma-Aldrich, cat# M1406; mouse anti-millimark pan neuronal marker, 1:1,000, Millipore, cat# MAB2300; mouse anti-rat CD68 (ED1), 1:100, Serotec, cat# MCA341R, mouse anti-Ox6 (MHCII), 1:500, Abcam, cat# Ab23990; rabbit anti-CNPase, 1:500, Cell Signaling, cat# 5664). Slides were incubated at room temperature for 1 h prior to being placed at 4°C overnight.

The following day, sections were washed in PBST and placed in blocking solution containing secondary antibodies (donkey anti-mouse AlexaFluor488, Jackson Laboratories, cat# 715-545-150; donkey anti-rabbit AlexaFluor594, Jackson Laboratories, cat# 711-585-152; donkey anti-rabbit AlexaFluor488, Jackson Laboratories, cat# 711-545-152; horse anti-mouse biotinylated, Vector Laboratories, cat# BA-2000) at a concentration of 1:250. Sections incubated with biotinylated secondary antibodies were washed in PBST then incubated in streptavidin bound Cy2 or AlexaFluor594 (Jackson Laboratories, 1:1,000; cat# 016-220-084 and cat# 016-580-084, respectively). All slides were then washed in PBST and dipped in Hoechst (1:1,000; Invitrogen) before being cover slipped.

For analysis, the region of interest was defined as the cortical area encapsulating the majority of rod microglia. In all cases, this region of interest was restricted to the primary sensory cortex. The trains (coupled microglia) were identified with the appropriate filter and imaged. Subsequently, the same field was imaged with filters for additional fluorophores. The areas of interest were first screened using an Olympus AX80 fluorescent microscope with motorized stage and attached DP70 digital camera. Fluorescently labeled sections were then photographed with an Olympus DSU confocal microscope with attached Hamamatsu digital camera and Slidebook software (Intelligent Imaging Innovations Inc.; Denver, CO, USA). Images were then de-convolved using Image-Pro 7 (Media Cybernetics Inc.; Bethesda, MD, USA). Analysis was conducted independent of hemisphere.

### Objective quantification of microglial alignment using fast Fourier transformation

Methods for quantification of alignment followed detailed methods published elsewhere [21]. Briefly, in Adobe Photoshop CS5, a 1,000 pixel diameter circle feather mask was applied to each photomicrograph. In NIH Image J the angle tool was used to measure the angle of interest of microglial alignment.

A fast Fourier transformation (FFT) image was generated, whereby the photomicrograph is transformed into a frequency domain based on the pixel intensity. Adjacent pixels in a specific orientation of the original photomicrograph are mirrored in the FFT image. A ring analysis with a radius of 700 pixels was applied to the center of the FFT image, producing radial sum intensity values for 360 radii around the circumference of the applied ring. The radial sum intensity values were used to generate the ratio from orthogonal using the calculation below [21]. The ratio from the orthogonal angle is used to determine alignment.

RMS=∑i=1360∟Ai2360

Ave∟M=∑j=122∟MjRMS22

Ratio to the mean orthogonal angle=

∑k=122∟NkRMSAve∟M22×100−100

Where,

RMS = Root mean square

∟A = The radial sum intensities of all 360 angles

∟M = The radial sum intensities of 22 angles, including the orthogonal angle ± 5 flanking angles and the opposite orthogonal angle ± 5 flanking angles

∟N = The radial sum intensities of 22 angles, including the angle of interest ± 5 flanking angles and the opposite angle of interest ± 5 flanking angles

The ratio from orthogonal was averaged within three sections from each animal, averaged within groups and compared between sham and injured animals by one-way ANOVA, with a Student-Newman-Keuls multiple comparison test *post-hoc* analysis. Significance was set at *P* <0.05. A value of 0 means no alignment, as this value increases so does the level of alignment.

## Results

### Microglial trains are predominantly stationed in the diffuse-injured primary sensory cortex

**Figure 1:**
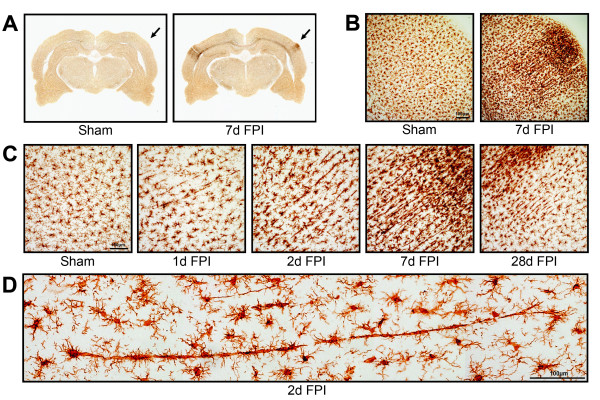
**Microglia alignment in Iba1 stained tissue from the primary sensory cortex after diffuse brain injury.** (**A**) Iba1 immunoreactivity is apparent in the primary somatosensory barrel field (S1BF) region of the cortex of midline fluid percussion injured animals (mFPI). The arrows mark principal regions of microglial hyper-intensity in the 7 day post-injury animals; sham animals show no appreciable microglia activation. (**B**) At higher power cellular pathological processes result in microglial hyper-intensity and morphological activation at 7 days post-injury, but not in uninjured sham animals. (**C**) The time course of microglial activation at the site of hyperactivity shows peculiar morphological alignment of microglia in the S1BF region at 1, 2, 7 and 28 days post-injury. At 1 day post-injury, microglia morphology shows reduced ramification with swollen or stretched cell bodies. These morphological changes are more evident at 2 days post-injury. By 7 days post-injury, microglia are elongated perpendicular to the dural surface across cortical layers, with morphology reminiscent of rod microglia. At 28 days post-injury, microglia alignment is reduced and morphology begins to regain its ramification. Maximal morphological alignment appears to be at 7 days post-injury. (**D**) Montage of photomicrographs showing a train of at least 10 aligned microglia 2 days post-injury.

Ramified microglia were evident in all regions of the sham-injured rat brain (Figure 1A). These cells had long, thin processes and were evenly scattered throughout the cortex (Figure 1B). Following diffuse brain injury, microglia became activated and their positioning was no longer evenly spaced. Of particular interest were the morphological changes of the microglia located in the primary sensory cortex, especially the primary somatosensory barrel field (S1BF). The Iba1-positive cell bodies became elongated and their processes prominently projected from the apical and basal ends rather than evenly from around the entire cell body, giving a rod-like morphology. At 1 day post mFPI, these cells began to align end-to-end, coupling to form trains (Figure 1C). These trains consisted of multiple cells, which traversed cortical layers and ran perpendicular to the dural surface. An extreme example is shown with over 10 rod microglia coupled that span 900 μm of cortex (Figure 1D). By day 7, the trains had become more pronounced with activated microglia also accumulating within the S1BF. By day 28, the trains of rod microglia had begun to depart. It is important to note that highly polarized trains of rod microglial cells were never observed in sham animals.

### Quantification of microglia alignment by fast Fourier transformation

**Figure 2:**
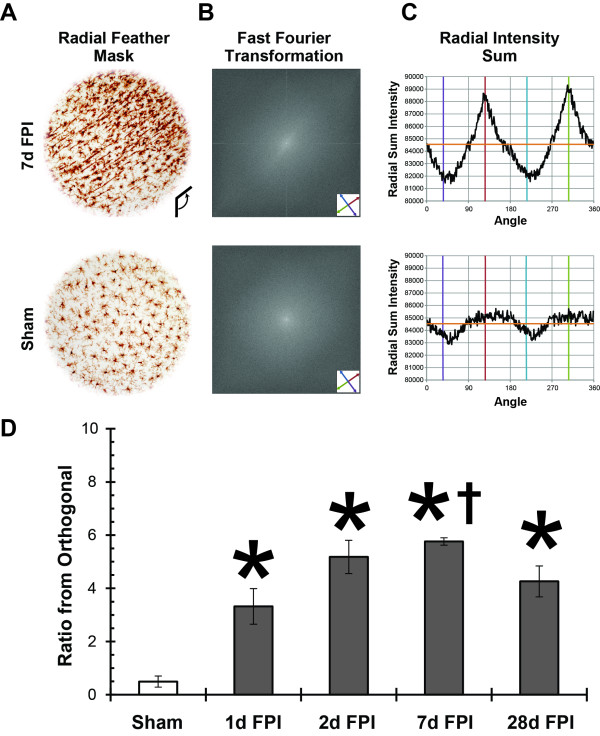
**Objective quantification of microglial alignment was performed on Iba1-stained tissue from the S1BF region using a fast Fourier transform (FFT) method.** (**A**) Representative images where radial feather mask was applied to eliminate edge effects. (**B**) Photomicrographs were then subjected to FFT using NIH Image J software; a ‘starburst’ effect can be seen in the 7d post-FPI image (oriented along the axis defined by the red and green arrows) which corresponds to the microglial alignment. (**C**) A ring analysis was applied to the FFT image and radial sum intensity value for each angle was calculated around the circumference from the center, as plotted in the histogram. For illustrative purposes, the orange horizontal line represents the root mean square of the radial sum intensities; the red and green vertical lines represent the angle of interest and opposite angle of interest, respectively; the purple and cyan vertical lines represent the orthogonal and opposite orthogonal angles, respectively. (**D**) Analysis was conducted for uninjured and brain-injured rats at various time points after diffuse brain injury (*n* = 3/group). At 1, 2, 7, and 28 days post-injury, the ratio from orthogonal was significantly higher than the uninjured sham values (*P <0.05, one-way ANOVA) and the ratio from orthogonal was significantly higher at 7 days post-injury compared to 1 day post-injury (†, *P* <0.05, one-way ANOVA). Mean ±SEM.

Objective quantification of microglial alignment was performed on Iba1-stained tissue from the S1BF region using a FFT method. To eliminate edge effects, a radial feather mask was applied to each photomicrograph in Adobe Photoshop CS5 (Figure 2A). Photomicrographs were transformed into a Fourier space using NIH Image J software, which produced the FFT image (Figure 2B). A ring analysis was then applied to the FFT image and a radial sum intensity value for each angle was calculated around the circumference from the center, as plotted in the histogram (Figure 2C). The histogram from a representative 7 day brain-injured animal shows the alignment of microglia by peaks in the radial sum intensity at the angle of interest (126°) and the opposite angle of interest (306°). On the other hand, the histogram from a representative sham animal does not show peaks or troughs of any magnitude. The ratio from orthogonal was calculated using the radial sum intensity values flanking the angles of interest as a fraction of the radial sum intensity values flanking the orthogonal angles, relative to the root mean square of the radial sum intensities [21]. A value of 0 indicated no alignment; however when the level of alignment increased so did the value (Figure 2D). A value of 0.5 was calculated for sham animals which indicated no appreciable microglia alignment. Following injury, the ratio from orthogonal at all post-injury time points was significant compared to sham (F_(4,10)_ = 17.15, *P* = 0.0002; one-way ANOVA). Although alignment was present at all time points following injury, it was most evident at day 7 as indicated by a significant increase from day 1 post-injury. With the maximal alignment observed at day 7, we elected to study day 7 post mFPI further.

### Rod microglial cells couple together to form trains in the S1BF

**Figure 3:**
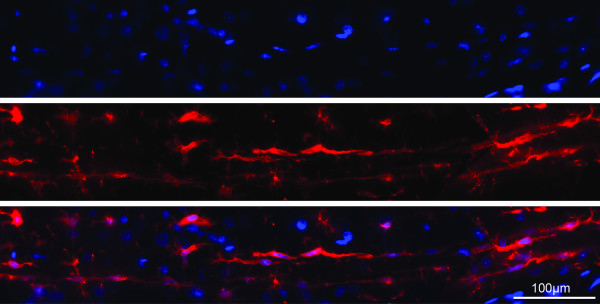
**Rod microglia couple together to form trains.** Immunofluorescence labeling of Iba1-positive microglia (red) with the nuclear marker Hoechst (blue) revealed that aligned rod microglia at 7 days post-injury were composed of multiple cells aligned in a single trajectory with their highly polarized processes seemingly intertwined. These rod microglia coupled together to form long trains which spanned multiple cortical layers.

Further analysis of the architecture surrounding rod microglia trains was undertaken at day 7 post mFPI; the time point shown to have the greatest cellular alignment. Unlike radial glial cells, which exhibit a single cell body with long projections [22-26], the rod microglia have coupled to form trains consisting of more than one rod microglia cell, as illustrated in Figure 3. We use the term coupling to indicate a physical proximity between the apical and basal processes of adjacent rod microglia. Adjacent rod microglia were identified by Hoechst positive staining of separate nuclei.

### Intermittent staining of carriages with other known markers for activated microglia

**Figure 4:**
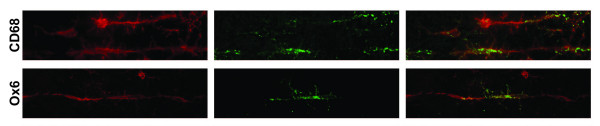
**Rod cells differentially express known markers for activated microglia.** Seven days post-injury Iba1^+^ rod microglia (red) show sporadic, punctate, intracellular staining for CD68 (ED1; green). Rod microglial trains intermittently co-localize with the MHC-II immunological marker (green), where some carriages in the train stained with Ox6 but adjacent ones did not.

Differences in microglial morphology have been discriminated by the expression of immunohistochemical markers. Therefore, immunohistochemical analysis of Iba1-positive rod microglia was undertaken with various known markers of activated microglia (Figure 4). After injury, punctate CD68 staining was observed in microglia throughout the brain. With respect to rod microglia, this sporadic staining was evident in each rod microglia along the train. The MHCII marker, Ox6, stained occasional microglia throughout the injured brain. When Iba1-positive trains were analyzed, intermittent carriages were OX-6 positive.

### Other glia do not align with trains of microglia

**Figure 5:**
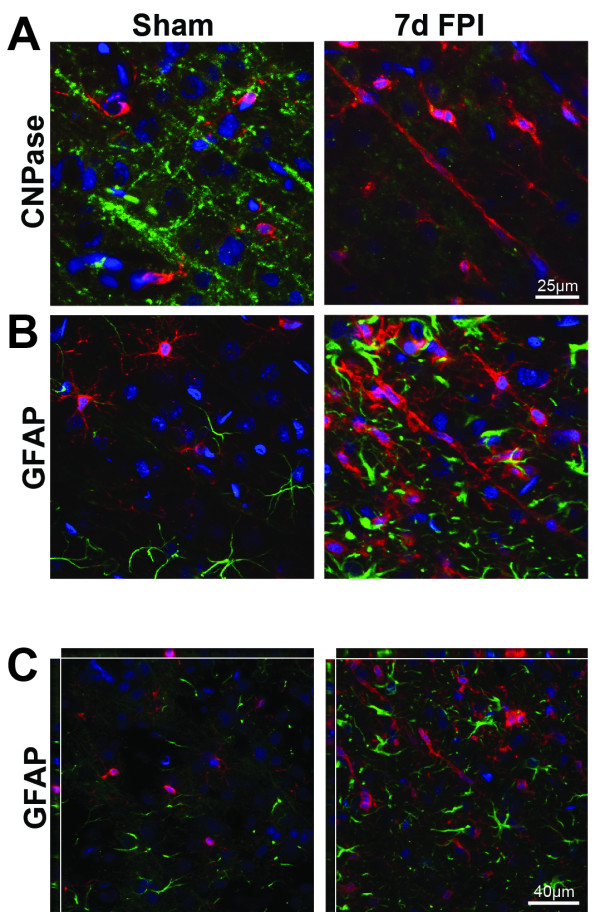
**Stations do not involve glial cells.** (A) In sham-injured rats, only ramified microglia (red) were observed with no association to normal CNPase stained oligodendrocytes (green). In TBI, there was evidence of decreased CNPase staining intensity at day 7 post TBI when rod microglial cells were present (Hoechst nuclei staining blue). (B) GFAP-positive astrocytes (green) were present amongst ramified microglia (red) in sham-injured animals. Following injury, astrocytes became hypertrophic and appeared in conjunction with rod microglia. Astrocytes showed no alignment with trains of rod microglia. (C) Confocal analysis determined that there was no co-localization of GFAP-positive astrocytes with rod microglial cells.

Double-labeling immunohistochemistry was performed with Iba1 and markers of oligodendrocytes (CNPase) or astrocytes (GFAP; Figure 5). CNPase staining was evident throughout the cortex of sham-injured animals; however at 7 days post mFPI, CNPase intensity was decreased in regions with trains of rod microglia (Figure 5A). Furthermore, there was no evidence of ingested myelin by rod microglial cells. Although astrocytes became activated after diffuse brain injury, there was no clear alignment of these cells with trains of rod microglia (Figure 5B). As expected, there was no co-localization of GFAP with microglia (Figure 5C).

### Trains of rod microglia associate with neuronal processes

**Figure 6:**
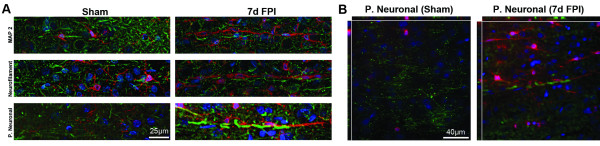
**Neuronal processes are the tracks for rod microglial trains.** Double-labeling of microglia (Iba1; red) with neuronal structural markers (green) and Hoechst nuclear dye (blue). (**A**) Ramified microglia (Iba1; red) in uninjured sham brain showed no association with MAP-2, neurofilament, or the pan-neuronal marker. Rod microglia in the sensory cortex 7 days post-injury were in close apposition with dendrites (MAP-2) and axons (neurofilament). (**B**) Additional staining with a pan neuronal marker clearly demonstrated the proximity of trains of rod microglia to neuronal structures via confocal analysis.

Visualization of dendrites and axons, as well as the complete neuronal process structure established the alignment of rod microglial trains parallel and adjacent to neuronal elements (Figure 6). In sham-injured animals only ramified microglia were observed.

Brain-injured animals displayed less MAP2 staining than sham-injured animals. There were often trains of microglia adjacent to long dendrites. No evidence of co-localization between MAP2 and Iba1 was observed.

Trains of rod microglia were also observed in close proximity to axons, without co-localization of these markers (Iba1 and neurofilament). Rod microglial trains were stationed adjacent to axons and dendrites with minimal gaps between the two. Lastly, we conducted staining with a pan-neuronal marker to label dendrites, axons, and cell bodies. Similar to MAP2 and neurofilament staining, neuronal elements of the pan-neuronal marker were adjacent to rod microglia trains, without co-localization. These data suggest that trains of rod microglia align closely to neuronal elements.

## Discussion

In the present communication, we explored the curious morphology of rod microglia to expand on the limited understanding of their role in neuropathology. Here we take advantage of the reproducibility and large quantity of rod microglia produced in the cortex after experimental diffuse TBI. Rod microglial cells within the S1BF were most numerous at 7 days post-injury, where they align perpendicular to the dural surface post-injury. Based on the results from the CD68 and OX-6 staining, rod microglial cells are immunophenotypically distinct from other microglial morphologies. It had been expected to observe co-localization of rod cells with a subset of immune markers. This was the case with CD68; however, the discontinuous labeling of OX-6 suggests a further division of rods that warrants investigation. Trains of rod microglia were associated with neuronal elements rather than other glial cells. These data support reports dating back a century ago, which illustrated the existence of rod-like cells adjacent to neuronal processes. Moreover, our results add support to the more recent hypothesis that microglia play a critical role in the recovery from CNS injury (reviewed in [27]).

The appearance of microglia has been reported to reflect their function [28,29]. Under normal physiological conditions microglia have a small, somewhat elongated cell body with long, fine processes. These ramified microglia are rather evenly spaced throughout the brain, with their processes pervading the entire brain. It is generally accepted that ramified microglia constantly survey the CNS and synapses for intruders/stresses which may disrupt structure and function of neuronal circuits [30,31]. This is highlighted by the observation that areas with fewer synapses have fewer microglia, such as white matter [32].

Various stimuli can rapidly activate morphological change in microglia. Cellular processes thicken and begin to retract from their surroundings [29]. At this stage these cells produce molecules with known immunological functions [28]. Further along the lines of activation, microglial cell bodies become more amoeboid and their processes thicken further and decline in number. These cells are presumed to be motile and secreting cytokines, with the ability to move to the site of injury/insult to reduce damage [28,29]. Migrating microglia typically show an asymmetrical morphology, some suggesting that they have a leading and a trailing edge [33]. These microglia differ from the ones described in the current communication as these rod cells appear symmetrical and are mainly found in the cerebral cortex. In cases where tissue damage is extensive, microglia become fully activated and at this point they are morphologically indistinguishable from infiltrating macrophages. Fully activated microglia have an amoeboid cell body and fully retracted processes. Functionally, these cells are thought to phagocytose debris/invading pathogens [28,29]. In focal TBI, active and amoeboid microglia are found throughout the contused or cavitated tissue and thought to promote tissue degeneration and contribute to repair [8,11,13-15,27,34]. In stark contrast, after diffuse TBI we found no amoeboid microglia/macrophages; nor evidence for ingested myelin or synaptic debris. Activated and rod microglia more likely contribute to the neuropathology and repair of injured circuits after diffuse TBI then the scavenging of debris.

Although the precise mechanism and the role of rod microglia coupling and train formation are yet to be identified, these cells are always observed to be end-to-end and never perpendicular to each other. The signals required to send microglia towards a rod morphology rather than a more traditional activation state were beyond the scope of the current study. Rod cells could be formed by migrating or proliferating microglia; not withstanding these cells ultimately align to neuronal elements. Indeed, microglia have been reported to migrate towards and adhere to damaged but surviving neurons, which allows for efficient uptake of diffusible molecules [28]. It is therefore plausible to suggest that rod microglia align to either less injured neurons or axotomized axons in an effort to limit damage. Alternatively, trains of rod microglia could just as easily align to protect uninjured axons. Clearly, rod microglia align with dendrites and axons of neuronal elements as described by Nissl over a century ago [1]. Co-labeling for trains of rod microglia and neuropathology would clarify reasons and associations of this microglia phenotype. However, we are technically limited at this time. Silver degeneration staining is incompatible with Iba1 immunohistochemistry. Double-labeling with the amyloid precursor protein (APP) marker of axonal injury was not pursued, because the antigen presentation is transient after experimental TBI, being absorbed by 72 h post-injury [12,35]. Future studies on a comprehensive neuropathological time course will include APP staining at earlier time points post-injury.

In our model, diffuse brain injury leads to perisomatic axotomy and argyrophilic localized to the S1BF over 7 days post-injury [12,36]. The rod microglia morphology was most evident at day 7 post TBI, coinciding with the highest levels of neuropathology [36]; rod microglia may align with these damaged axons. Furthermore, rod microglial cells were observed concomitantly with reduced staining of CNPase indicating a loss of myelin detection. Cascades of neurodegenerative and regenerative events may hit a crossroad at day 7, which catalyzes the maximal coupling of rod microglia. We believe rod microglia are contributing to circuit reorganization in the wake of neuropathology; the detrimental and beneficial effects of which are yet to be determined.

Here, we demonstrate for the first time a preponderance of rod microglia following experimental diffuse TBI. At this time, it remains unclear whether higher or lower levels of injury would alter the number or duration of rod microglia. Previous studies have proposed that rod microglia only form in tissue which is devoid of cavitation and contusion [4], suggesting that experimental models of focal TBI and stroke would result in an unfavorable environment for rod-microglia formation. Indeed, preliminary evidence indicated that rod microglia can be found in experimental blast-overpressure brain injury [37].

Some could question the existence of rod microglia, and presume the formation of trains to be radial glial cells. We however argue that this is not the case due to distinct morphological (Figures 1 and 3) and phenotypic differences (Figure 4). Radial glial cells are described as a single cell with long processes [22,23]. Additionally, reports indicate that radial glia will stain with a variety of markers including GFAP, S100β, and vimentin (reviewed in [23]). The rod microglial trains in the current study are composed of multiple cells as evidenced by the nuclear staining and they failed to co-localize with radial glial markers (S100β and vimentin, images not shown). The cell morphology described here is not one for radial glia, but rather a distinct microglia morphology, with one or more phenotypes.

It is intriguing to contemplate a functional role for the trains of rod microglia. One possibility is synaptic support, whereas synaptic stripping is equally likely [38]. Indeed, complementary work from our laboratory highlights a functional role for the S1BF (predominant location of rod microglia) in behavioral morbidity [36]. The experimental model of midline fluid percussion offers the opportunity to examine the diffuse injured brain in the absence of contusion and cavitation. It has been described as a model of circuit disruption rather than destruction [12,20]. Disrupted circuits reorganize [36,39] and mediate late-onset sensory sensitivity, where brain-injured rats exhibit whisker sensitivity (whisker nuisance task) that develops over 28 days post TBI [20]. The circuitry used by rats in the whisker nuisance task incorporates the S1BF, the region we have documented to have large numbers of rod microglia by 7 days post-injury. Taken together, these data implicate rod microglia in the development of late-onset morbidities. However, more work is required to clarify the precise role that rod microglia play in behavioral morbidity.

Mechanisms of microglia activation and the functional implication on neuronal circuit function remain understudied. Microglia influence the injury magnitude and recovery in neurotrauma patients through numerous mechanisms, including clearing of cellular debris, neurotoxicity, promoting and directing axon growth, or mediating the reorganization of neuronal circuit structures [40,41]. The role of microglia after injury has yet to be fully elucidated. However, depending on the activation stimulus (for example, APP or IL-4) the phenotype of the microglia may be neuro-toxic or -trophic [42]. Additionally, a well-controlled helper T-cell response induces the microglia to adopt a phenotype that facilitates neuronal protection and tissue repair [42-44]. Of note, resident microglia can play a role in tissue repair, similar to that described for resident macrophages in peripheral organs (reviewed in [45]). Furthermore, synergistic effects of microglia and astrocytes are needed for tissue remodeling after lesions, as they contribute to the re-establishment of the blood-brain barrier, as well as by secreting anti-inflammatory cytokines to suppress the inflammatory cascade [46].

## Conclusion

Extensive research has been conducted investigating the role microglia play in their various activation states; however, the rod-like morphology observed in the current study has received comparatively little attention. Rod microglial cells are defined by elongated cell bodies with processes that prominently project from the basal and apical ends, and are aligned to neuronal processes in cerebral cortex. We hypothesize that these cells play a role in neuronal circuit reorganization; however, their exact function is yet to be elucidated. Importantly, we have established a new model to analyze rod cell biology and their role in brain pathology.

## Competing interests

The authors have no competing interests to declare.

## Authors’ contribution

JMZ carried out tissue collection, sectioning and immunohistochemistry, data collection, analysis and interpretation as well as manuscript preparation. SET conducted immunohistochemical staining, image collection, analysis and compilation as well as FFT analysis and manuscript drafting. TC provided substantial contributions to the concept and design of the study including the generation of preliminary FFT analysis and manuscript draft. JLH contributed to image acquisition, technical and conceptual study elements, and manuscript revision. JL contributed concept and design of the study, intellectual input, interpretation of data, and manuscript revision. All authors have read and approved the final version of the manuscript.
